# Sodium channels as a new target for pain treatment

**DOI:** 10.3389/fphar.2025.1573254

**Published:** 2025-03-26

**Authors:** Rui Chen, Yiran Liu, Liu Qian, Mingliang Yi, Hong Yin, Shun Wang, Bingbing Xiang

**Affiliations:** ^1^ Department of Anesthesiology, Chengdu Fifth People’s Hospital (The Second Clinical Medical College, Geriatric Diseases Institute of Chengdu/Cancer Prevention and Treatment Institute of Chengdu, Affiliated Fifth People’s Hospital of Chengdu University of Traditional Chinese Medicine), Chengdu, China; ^2^ Nursing Department, Cujin Community Health Service Center, Chengdu, China; ^3^ Department of Anesthesiology, West China Hospital, Sichuan University, Chengdu, China; ^4^ Department of Anesthesiology, Clinical Medical College and The First Affiliated Hospital of Chengdu Medical College, Chengdu, China

**Keywords:** pain, sodium channels, drug discovery, Nav1.7, Nav1.8, Nav1.9

## Abstract

Voltage-gated sodium channels, especially the Nav1.7, Nav1.8, and Nav1.9 subtypes, play a crucial role in the transmission of pain signals. Nav1.7 is considered a threshold channel that regulates the generation of action potentials and is closely associated with various hereditary pain disorders. Nav1.8 primarily participates in inflammatory and neuropathic pain within the peripheral nervous system. Its characteristic of not involving the central nervous system makes it a potential target for non-addictive analgesics. Nav1.9 has shown significant involvement in cold pain sensing and small fiber neuropathy, although its mechanism of action is still under further investigation. Currently, despite promising results from preclinical studies, sodium channel inhibitors have not fully met expectations in clinical trials due to issues such as drug selectivity, dosing, and safety. The development of Nav1.7 and Nav1.8 inhibitors faces challenges such as drug intolerance, insufficient target occupancy, and off-target side effects. Future research may promote the development of non-opioid analgesics through combined inhibition strategies targeting multiple Nav subtypes, as well as improving drug selectivity and bioavailability. Overall, sodium channel inhibitors remain a key area of research in pain management, but their clinical application prospects still require further exploration.

## Introduction

Pain is a significant global public health issue that severely affects the quality of life of millions of individuals. Currently, the treatment of severe and chronic pain primarily relies on opioid analgesics. However, the side effects and addiction risks associated with these drugs limit their widespread use (The, 2021). Therefore, exploring safer and more effective pain management strategies is imperative. In recent years, with the advancement of molecular biology and neuroscience, voltage-gated sodium channels (VGSCs, also known as Nav) have been found to play a critical role in pain perception, transmission, and chronicity, making them a key focus of pain research. As a result, targeting specific subtypes of voltage-gated sodium channels offers a novel direction for pain treatment. This review provides an overview of the major voltage-gated sodium channel subtypes closely associated with pain and discusses their potential as therapeutic targets, aiming to offer safer and more effective alternatives for pain management.

## Structure and classification of sodium channels

In 1952, Hodgkin and Huxley (1952) first confirmed the existence of voltage-gated sodium channels and elucidated their key role in action potentials. Sodium ion channels primarily mediate the rapid influx of sodium ions during cell depolarization, thereby generating the rising phase of the action potential. Typical sodium ion channels are widely distributed in nerves, skeletal muscles, and cardiac muscles, facilitating the generation and conduction of action potentials (AP) in excitable cells, and playing critical roles in neural signal transmission and cardiac rhythm regulation (Payandeh and Minor, 2015).

Through electrophysiological recording, biochemical purification, and molecular cloning techniques, various types of voltage-gated sodium channels have been successfully identified. Voltage-gated sodium channels are a protein family composed of two or three subunits, with the pore-forming α subunit (260 kDa) linked to one or two β auxiliary subunits (30–40 kDa) (Catterall, 2000). The α subunit of the voltage-gated sodium channel in mammals is a 260 kDa protein that contains four homologous transmembrane domains (DI-DIV), each composed of six segments (S1-S6), which form α-helices that traverse the membrane. These segments, through the S5 and S6 helices and the P-loop that connects them, together form the channel pore (Ogata and Ohishi, 2002; England and De Groot, 2009). To date, nine functional members of this family have been described, each exhibiting similar predicted secondary structures (England and De Groot, 2009). In recombinant systems, a single α subunit can form a fully functional channel. Based on the genetic classification of sodium channel α subunits, they are named Nav1.1 to Nav1.9 and are encoded by SCN(X)A genes. Additionally, the 10th sodium channel protein, Nax, is not voltage-gated and is primarily involved in salt sensitivity (Ogata and Ohishi, 2002).

Four β subunits (β1-4) have been identified, believed to consist of a single transmembrane α-helix (Tseng et al., 2007). The C-terminus on the intracellular side is short, while the N-terminus on the extracellular side is larger and is thought to form an immunoglobulin-like domain, with a sequence similar to the cell adhesion molecule family (England and De Groot, 2009). β1 and β3 subunits covalently bind to the α subunit via disulfide bonds, whereas β2 and β4 bind much more weakly. For certain channels, β subunits play an important role in altering the biophysical properties of the channel and in the insertion of the α subunit into the plasma membrane (Tseng et al., 2007). Each sodium channel subtype exhibits unique biophysical properties. From a pharmacological perspective, sodium ion channels can be divided into two main categories: tetrodotoxin-sensitive (TTX-S) and tetrodotoxin-resistant (TTX-R) types. Nav1.1, Nav1.2, Nav1.3, Nav1.4, Nav1.6, and Nav1.7 are TTX-sensitive, while Nav1.5, Nav1.8, and Nav1.9 are TTX-resistant. The distinct distribution of different sodium channel subtypes in the nervous system, along with their sensitivity to drugs, makes them potential therapeutic targets. Specific distribution patterns are summarized in Table 1.

**TABLE 1:** Voltage-gated sodium channel nomenclature, chromosomal location and tissue distribution.

Formal name	Traditional names	Gene symbol	Chromosomal location (human)	Tetrodotoxin sensitivity	Major tissue expression	Expression in DRG	Effect of mutation
Nav1.1	Type I, rat I, Scn Ia, HBSCI, GPBI	SCN1A	2q24	S	CNS, PNS	+++	Epilepsy
Nav1.2	Type II, rat II, HBSCII, HBA	SCN2A	2q23–24	S	CNS, PNS	+	Epilepsy
Nav1.3	Type III, rat III	SCN3A	2q24	S	CNS (embryonic), PNS	upregulated after axotomy	None reported
Nav1.4	SkM1, u1	SCN4A	17q23–25	S	Skeletal muscle	-	Myotonia, periodic paralysis
Nav1.5	SkM2, rH1, H1	SCN5A	3p21	R	Heart	-	Long QT, Brugada syndrome, progressive familial heart block
Nav1.6	Type IV, NaCh6, Na6, PN4, Scn8a, CerIII	SCN8A	12q13	S	CNS, PNS, glia nodes of Ranvier	+++	Cerebellar atrophy
Nav1.7	PN1, hNE, Nas	SCN9A	2q24	S	PNS Schwan cell	+++	Increased and decreased pain sensitivity
Nav1.8	SNS, PN3, NaNG	SCN10A	3p21–24	R	PNS (sensory neurons)	+++	None reported
Nav1.9	SNS2, NaN, NaT, SCN12A	SCN11A	3p21–24	R	PNS	+++	Paroxysmal pain, small-fiber neuropathy
Navx	Nav2.1, Nav2.2, Nav2.3, SCL11, NavG	SCN6/7A	2q21–23	R	Heart, uterus, glia, PNS smooth muscle	+	-

CNS, central nervous system; PNS, peripheral nervous system; S, sensitive; R, resistant.

## Voltage-gated sodium channels and pain

Voltage-gated sodium channels (VGSCs) play a fundamental role in sensory conduction, particularly in neuronal excitation, the transmission of pain signals, and the regulation of pain. As a result, they have emerged as promising targets for novel analgesic drugs (Waxman, 2023). Voltage-gated sodium channels are essential for the generation of action potentials in neurons and are critical for neuronal excitability and signal transmission. They are expressed in multiple regions of the nervous system, including primary sensory neurons and dorsal root ganglia (DRG). As a long-established class of drug targets, VGSCs have been targeted by various drugs, including local anesthetics (e.g., lidocaine), classic anticonvulsants (e.g., lamotrigine, carbamazepine, phenytoin), and antiarrhythmic drugs (e.g., mexiletine, flecainide). These drugs exert their pharmacological effects by modulating sodium channels (England and De Groot, 2009). They have shown significant efficacy in treating clinical conditions of excessive neuronal excitability caused by abnormal sodium channel activity, such as epilepsy and neuropathic pain. Local anesthetics, in particular, are used for producing nerve blocks.

These drugs were discovered through empirical methods, typically in isolated tissue models designed to mimic specific clinical pathologies. For instance, drugs are often identified based on their pharmacological activity in heart or brain slice preparations, a method that has been successful in discovering drugs for treating arrhythmias or epilepsy (Borchard et al., 1982; Piredda et al., 1986). However, research specifically aimed at identifying molecular targets, such as voltage-gated sodium channels, was only completed years later, well after the discovery of these drugs, which predated the cloning and expression of sodium channel subtypes (Noda et al., 1986).

With the advancement of research technologies, clinical sodium channel modulators are now typically characterized through electrophysiological methods, which often reveal limitations in terms of selectivity and efficacy, with poor subtype selectivity. While empirical methods have led to the discovery of drugs in the past, the research challenges and standards we face today have significantly raised the bar. The current goal is to identify drugs that are subtype-selective, more efficient, and associated with fewer side effects, to meet the growing demand for safer and more precise treatments.

## The potential of voltage-gated sodium channels for pain treatment

Voltage-gated sodium channels (VGSCs) play a fundamental role in pain transmission, particularly in regulating neuronal excitability and participating in the transmission and modulation of pain signals. Therefore, sodium ion channels have become important targets in the development of novel analgesic drugs. VGSCs are essential for the generation of action potentials in neurons, crucial for neuronal excitability and signal conduction, and are widely expressed in various regions of the nervous system, including primary sensory neurons and dorsal root ganglia (DRG) (England and De Groot, 2009). The perception and maintenance of pain depend on the normal functioning of sodium ion channels, and directly targeting sodium channels in the peripheral nervous system holds the potential to avoid the central side effects commonly associated with traditional opioid drugs and other centrally acting medications. Thus, peripheral targeting of sodium channels in pain therapy holds significant clinical value.

Recent studies have shown that alterations in sodium ion channels are the molecular basis for peripheral sensitization, central sensitization, and disinhibition following inflammatory or neuropathic injury. These changes are also key mechanisms underlying the development of neuropathic pain (Bi et al., 2017; Bennett et al., 2019). The expression and function of sodium ion channels are thought to undergo long-term changes that contribute to the formation of chronic pain states, providing new targets and research approaches for the treatment of chronic pain and neuropathic pain (Sopacua et al., 2019). While this theoretical framework is reasonable, the widespread expression of many sodium ion channels in various tissues (such as the heart, skeletal muscles, and smooth muscles) increases the risk of off-target effects. This presents a complex challenge in developing selective sodium channel modulators that effectively target peripheral pain-sensing neurons while avoiding interference with other physiological functions.

Sodium ion channels are key regulatory factors in the pain transmission process, particularly the Nav1.7, Nav1.8, and Nav1.9 subtypes, which play crucial roles in transmitting peripheral pain signals to the central nervous system. As a result, these sodium channels are considered potential targets for pain treatment. Studies have shown that in acute inflammatory pain states, the gene expression of voltage-gated sodium channels is significantly increased, particularly for Nav1.7 and Nav1.8 (Bi et al., 2017; Liu and Wood, 2011). This overexpression leads to a reduced threshold for C-fibers and Aδ-fibers, resulting in intensified pain and the development of primary hyperalgesia. For Nav1.8 and Nav1.9, these channels play pivotal roles in inflammatory and neuropathic pain (Faber et al., 2012; Luiz et al., 2015). The expression of Nav1.8 is significantly increased in chronic inflammatory pain (Sopacua et al., 2019), while Nav1.9 is critically involved in cold sensation and the development of neuropathic pain (Lolignier et al., 2015). Therefore, inhibiting Nav1.7, Nav1.8, and Nav1.9 has become a crucial direction in pain therapy. Researchers are actively working to develop drugs that specifically target and inhibit these subtypes, aiming to achieve more precise and effective pain relief.

## Nav1.7 as a target for pain treatment

Nav1.7 is predominantly expressed in peripheral nociceptive neurons and various types of primary sensory neurons, including dorsal root ganglia (DRG), trigeminal ganglia (TG), olfactory epithelium, and sympathetic neurons (Toledo-Aral et al., 1997; Weiss et al., 2011; Morinville et al., 2007). Nav1.7 is considered a threshold channel that triggers action potentials, enhancing the probability of neurons reaching the depolarization threshold under subthreshold stimuli, thereby playing a crucial role in neuronal excitation (Cummins et al., 1998). The expression of Nav1.7 in most pain-sensing neurons is closely related to several hereditary pain-related disorders. For example, gain-of-function mutations in Nav1.7 can lead to chronic spontaneous pain or extreme pain symptoms, such as inherited erythromelalgia (IEM) (Meents et al., 2019; Greco et al., 2018), paroxysmal extreme pain disorder (PEPD) (Yuan et al., 2023), and small fiber neuropathy (SFN) (Mann et al., 2019; Eijkenboom et al., 2019). In contrast, loss-of-function mutations in Nav1.7 result in congenital insensitivity to pain (CIP), where patients are unable to perceive pain but remain sensitive to touch and pressure, often accompanied by anosmia (Rajasekharan et al., 2017; Drissi et al., 2020).

The role of Nav1.7 as a target for pain management has been supported by genetic evidence from congenital insensitivity to pain, providing a strong theoretical foundation for the discovery of Nav1.7-targeted drugs and offering new insights into the precision treatment of pain. For a long time, the lack of high-resolution three-dimensional structural data limited the deep understanding of the conformational mechanisms of Nav1.7 and the development of Nav1.7-targeted drugs. However, in 2022, Huang et al. (Huang et al., 2022a; Huang et al., 2022b) used cryo-electron microscopy (cryo-EM) for the first time to analyze the high-resolution structure of hNav1.7, providing critical insights into the mechanistic coupling of voltage-gated sodium ion channels and molecular pathological explanations for mutations associated with pain. Subsequently, researchers analyzed the high-resolution cryo-EM structures of Nav1.7 in complex with peptide toxins and representative small-molecule inhibitors, laying the foundation for the design and optimization of Nav1.7-targeted drugs (Huang et al., 2022a; Zhang et al., 2022).

In July 2013, the U.S. FDA approved vixotrigine as an orphan drug for the treatment of trigeminal neuralgia. Functional analysis revealed that vixotrigine did not affect the activation properties of Nav1.7 but promoted its inactivation by stabilizing the inactivated state, delaying recovery from inactivation, thereby inhibiting Nav1.7 channel activity (Zheng et al., 2018). However, despite showing some efficacy in early clinical trials, its phase II clinical study was terminated due to failure to meet primary or secondary efficacy endpoints (Alsaloum et al., 2020). Pfizer’s PF-05089771, a highly selective Nav1.7 inhibitor (Alexandrou et al., 2016), interacts with the voltage-sensing domain (VSD) of the channel, stabilizing the inactivated conformation of Nav1.7 and inhibiting the depolarized Nav1.7 channel (Theile et al., 2016). However, in a randomized double-blind clinical study for diabetic neuropathic pain, the drug failed to significantly improve pain scores, leading Pfizer to discontinue its development (Bankar et al., 2018; Eagles et al., 2022).

The development of Nav1.7 inhibitors as pain management drugs has faced numerous challenges in translating from preclinical research to clinical trials. Despite the development of several highly selective and high-affinity Nav1.7 inhibitors, most have shown poor analgesic efficacy in both preclinical pain animal models and human clinical trials. Chronic pain, which is the most common type of pain in clinical settings, involves complex pathophysiology where Nav1.7 is modulated by multiple factors, such as SUMOylation and PKC phosphorylation, which differ significantly from the characteristics of Nav1.7 observed in acute, single-dose preclinical animal models (Moutal et al., 2020; Dustrude et al., 2016; Kerth et al., 2021).

Research by Macdonald et al. (2021) highlighted an intriguing finding: although peripheral deletion of Nav1.7 leads to extreme insensitivity to pain, the activity of nociceptors (pain receptors) was not affected by the absence of Nav1.7. Further studies revealed that synaptic inputs to the dorsal horn might be influenced by opioid-dependent mechanisms. Moreover, blocking central opioid receptors could reverse the analgesic effects in both Nav1.7-deficient mice and humans. This research provides a biological explanation for why peripheral Nav1.7 inhibitors fail to induce pain relief, suggesting that the central terminals of Nav1.7 and associated opioid signaling pathways could represent alternative therapeutic targets for future pain relief strategies.

These findings emphasize that while targeting peripheral Nav1.7 may not yield the desired analgesic effects in some cases, focusing on central mechanisms involving both Nav1.7 and opioid signaling could offer a promising avenue for more effective treatments for chronic pain. Thus, the challenge moving forward is not only to refine the selective targeting of Nav1.7 but also to consider how central and peripheral nervous system interactions influence pain processing and treatment outcomes.

## Nav1.8 as a pain treatment target

While Nav1.7 has been a key target for pain relief research due to its strong genetic validation in humans, clinical trials involving various Nav1.7 inhibitors have not been very successful. Key issues include insufficient effectiveness on spinal distributions, inadequate target binding, and poor bioavailability, which have hindered the practical application of Nav1.7 inhibitors. Additionally, challenges in clinical trial design and the selectivity of the target have made progress difficult. As a result, Nav1.7 has been somewhat overshadowed, and research attention has shifted towards Nav1.8, a promising target for pain therapy.

Nav1.8 plays a critical role in pain signal transduction and is predominantly expressed in peripheral nociceptive neurons. It is a tetrodotoxin-insensitive sodium channel (Waxman and Zamponi, 2014), meaning it is less susceptible to inhibition by substances like tetrodotoxin, which blocks most voltage-gated sodium channels. Unlike Nav1.7, which is involved in the central nervous system, Nav1.8 is primarily located in peripheral sensory neurons and is not involved in central nervous system activities. This characteristic makes Nav1.8 inhibitors potentially effective at relieving pain without the risk of addiction or negative effects on motor function, which are common concerns with central nervous system-targeting drugs (Waxman, 2023; Wood et al., 2002). Therefore, Nav1.8 is considered a highly selective and promising pain treatment target.

Nav1.8 is mainly expressed in peripheral pain receptors, including small-diameter neurons in the dorsal root ganglion (DRG), nodal neurons, and trigeminal ganglion neurons (Wood et al., 2002). In primary sensory neurons, the opening of Nav1.8 channels leads to a large influx of Na + ions, resulting in membrane depolarization and playing a critical role in generating and maintaining action potentials (Waxman and Zamponi, 2014; Renganathan et al., 2001). Specifically, while Nav1.7 regulates the threshold for action potential firing, Nav1.8 mainly influences the rising phase and amplitude of the action potential (Cummins et al., 1998; Mcdermott et al., 2019).

Nav1.8 is closely associated with various pain types, including inflammatory pain and neuropathic pain. Studies have found functional mutations in the SCN10A gene encoding Nav1.8 in patients with chronic pain from small fiber neuropathy (Sopacua et al., 2019). Although the loss-of-function mutations of Nav1.8 have not been fully described in human diseases, evidence from animal models suggests that Nav1.8 plays a critical role in both inflammatory and neuropathic pain (Lv et al., 2023). Gain-of-function mutations in Nav1.8 lead to excessive neuronal excitability and inappropriate spontaneous discharges, which could be a cause of some painful peripheral neuropathies (Faber et al., 2012).

Thus, Nav1.8 represents a promising and selective target for the development of novel pain relievers, particularly for chronic pain, as its inhibition appears to provide effective pain relief without the typical side effects such as addiction or motor dysfunction. This makes it an attractive alternative to traditional pain treatments and a focal point for ongoing drug development in the pain management field.

VX-150 was one of the first compounds developed by Vertex Pharmaceuticals targeting the Nav1.8 sodium channel. In multiple proof-of-concept Phase II clinical trials, VX-150 demonstrated promising therapeutic effects (Hijma et al., 2021). However, despite its initial success, the clinical application of VX-150 was limited by a relatively high required dosage and associated adverse effects, such as dizziness and headaches, leading to the cessation of its further development. Nevertheless, the research on VX-150 has provided substantial scientific evidence supporting the viability of Nav1.8 as a potential analgesic target.

Additionally, several selective Nav1.8 blockers, such as A-803467 and PF-01247324, have shown significant analgesic effects in models of neuropathic and inflammatory pain (Payne et al., 2015; Jarvis et al., 2007). Building on a deep understanding of the biological mechanisms of Nav1.8 and the accumulation of extensive data, Vertex Pharmaceuticals has developed an innovative molecule, VX-548, which has achieved significant progress in clinical stages. As a selective Nav1.8 inhibitor, VX-548 was designed specifically for the treatment of moderate to severe acute pain, demonstrating favorable pharmacokinetic properties and safety profiles, with reduced dosing requirements (Jones et al., 2023).

Reportedly, the FDA is currently reviewing the regulatory approval for Vertex’s small molecule drug suzetrigine (VX-548) for the treatment of moderate to severe acute pain. If approved, this would be the first new pain medication to reach the market in decades (Kingwell, 2024). However, on 19 December 2024, Vertex disclosed the results of VX-548’s Phase II study for sciatica (LSR), revealing that although VX-548 significantly reduced pain in the trial, similar pain relief was also observed in the placebo group. This raised concerns regarding the quality of the trial data and the future prospects of the drug, with the public perceiving its performance to be well below expectations.

## Nav1.9 as a pain treatment target

Nav1.9 is primarily expressed in endogenous muscle and visceral sensory nociceptors, small-diameter dorsal root ganglion (DRG) neurons (diameter <30 μm), and trigeminal ganglia, among other regions (Brackx et al., 2023). Unlike other sodium channels, Nav1.9 exhibits slow activation and deactivation kinetics, generating sustained Na + currents, and is likely independent of the depolarizing phase of the action potential (Dib-Hajj et al., 2015; Dib-Hajj et al., 1999). The inward sodium ion current mediated by the Nav1.9 channel plays a crucial role in amplifying subthreshold stimuli and modulating the resting membrane potential of cells (Dib-Hajj et al., 2015). Although the precise role of Nav1.9 in pain remains controversial, it is generally recognized as playing a critical role in the initiation of nociceptive signals and the development of hyperexcitability in neurons following inflammation (Dib-Hajj et al., 2015; Wu et al., 2018).

The SCN11A gene encodes the primary functional α subunit of the human Nav1.9 channel. Mutations in SCN11A have been associated with several hereditary pain disorders, including familial episodic pain, congenital insensitivity to pain, and inherited small fiber neuropathy (Leipold et al., 2015; Contijoch Roqueta et al., 2020). In Nav1.9 knockout mice, studies have shown diminished inflammatory responses and a significant reduction in mechanical hypersensitivity induced by CFA and formalin (Lolignier et al., 2011). These findings suggest that Nav1.9 plays an essential role in the pathogenesis of pathological pain, with gain-of-function mutations potentially causing abnormal neuronal excitability and leading to excessive pain discharge.

Further studies by Lolignier et al. have confirmed the pivotal role of Nav1.9 in both normal and pathological cold pain signaling, proposing that Nav1.9 acts as a subthreshold amplifier of cold sensory signals (Lolignier et al., 2015). Disruption of Nav1.9 expression in rodents has been shown to increase the threshold for cold-induced pain, further supporting its involvement in cold nociception. Due to the relatively low sequence homology (approximately 50%) between Nav1.9 and other Nav channel subtypes, it is relatively straightforward to develop selective inhibitors targeting Nav1.9, with theoretically fewer side effects. This makes Nav1.9 a highly promising target for pain treatment.

## Challenges and prospects of selective sodium channel inhibitors

The development of selective sodium channel inhibitors could revolutionize non-opioid pain management. However, the development of subtype-selective sodium channel inhibitors remains a significant challenge. Sodium channels play a crucial role in many physiological processes, particularly in the excitability of cardiac and neuronal cells, making their highly conserved nature a potential safety concern. A review of the development of selective sodium channel inhibitors reveals that although several high-selectivity, high-affinity inhibitors for Nav1.7 and Nav1.8 have been developed, clinical trial results have shown that voltage-gated sodium channel subtype-selective inhibitors have not fully achieved the expected analgesic effects. The reasons for this remain unclear.

Researchers speculate that the dynamic changes of different Nav channel subtypes on nociceptors might be a key factor contributing to the suboptimal performance of selective voltage-gated sodium channel subtype inhibitors. A recent preprint study published in eLife suggests that nociceptors can achieve similar excitability through different Nav channel subtypes, which is crucial for the overall excitability of neurons and their physiological function (Xie et al., 2024). The dynamic shifts in Nav subtypes may limit the effects of certain drugs under specific pathological conditions, emphasizing the need to consider these changes when developing inhibitors targeting Nav subtypes. Moreover, MACDONALD DI et al. demonstrated that although NaV1.7 knockout mice show pronounced analgesic effects, nociceptor activity in the dorsal root ganglia (DRG) remains largely unaffected (Macdonald et al., 2021). Simultaneously, opioid receptor blockade can reverse the analgesic effects in NaV1.7 knockout mice and humans, suggesting a profound analgesic synergy between NaV1.7 inhibitors and low-dose opioids (Deuis et al., 2017; Emery and Wood, 2019).

## Conclusion and outlook

Therefore, future pain treatment strategies may not rely solely on a single sodium channel inhibitor but could involve the combination of selective inhibitors targeting multiple Nav subtypes (such as NaV1.7, NaV1.8, and NaV1.9) to more effectively block pain transmission pathways. Additionally, considering the dynamic changes in Nav subtypes on nociceptors, the combination of low-dose opioids or other analgesic mechanisms may further enhance efficacy while mitigating side effects, such as dependency.
